# Metagenomic next-generation sequencing in a diagnosis of *Pneumocystis* pneumonia in an X-linked immunodeficient child: a case report

**DOI:** 10.3389/fped.2023.1183601

**Published:** 2023-06-26

**Authors:** Lu Qing, Yufei Zhao, Ye Zhang, Yuanlin Guan, Guoyan Lu

**Affiliations:** ^1^ Key Laboratory of Birth Defects and Related Diseases of Women and Children of MOE, Department of Pediatrics, West China Second University Hospital, Sichuan University, Chengdu, China; ^2^ Department of Pediatrics, The Third Hospital of Mianyang, Sichuan University, Mianyang, China; ^3^ Department of Scientific Affairs, Hugobiotech Co., Ltd., Beijing, China

**Keywords:** metagenomic next-generation sequencing, *Pneumocystis jirovecii* pneumonia, X-linked immunodeficiency, child, diagnosis

## Abstract

**Background:**

The diagnosis of *Pneumocystis* pneumonia (PCP) remains challenging in certain specific clinical situations. Metagenomic next-generation sequencing (mNGS), as a novel diagnostic method, may help in the diagnosis of PCP.

**Case presentation:**

A 6-month-old male child developed acute pneumonia and sepsis. This child had previously suffered from *Escherichia coli* septicemia and was cured. However, the fever and dyspnea relapsed. Blood tests revealed a low lymphocyte count (0.69 × 10^9^/L) and acute inflammatory markers such as high-level procalcitonin (8.0 ng/ml) and C-reactive protein (19 mg/dl). Chest imaging showed inflammation and decreased translucency in both lungs but no thymus shadow. Various serology tests, the 1,3-beta-D-glucan test, culture, as well as sputum smear failed to detect any pathogens. mNGS with blood helped identify 133 specific nucleic acid sequences of *Pneumocystis jirovecii*, suggesting an infection with this pathogen. After treatment with trimethoprim-sulfamethoxazole for 5 days, the patient's condition improved, but the child still needed ventilator support. Unfortunately, the child died soon after because of respiratory failure after his parents decided to abandon treatment. The family declined an autopsy on the child, and therefore, an anatomical diagnosis could not be obtained. Whole-exome sequencing suggested X-linked immunodeficiency. A hemizygous mutation of c.865c > t (p.r289*) was detected in the *IL2RG* gene, which was inherited from the mother (heterozygous state).

**Conclusion:**

This case report highlights the value of mNGS in diagnosing PCP when conventional diagnostic methods fail to identify the agent. Early onset of recurrent infectious diseases may indicate the presence of an immunodeficiency disease, for which timely genetic analysis and diagnosis are crucial.

## Introduction

*Pneumocystis* pneumonia (PCP) is a major opportunistic infection in immunocompromised patients. Thus far, it has been found that the pathogen of human PCP, *Pneumocystis jirovecii*, remains incapable of growing *in vitro*. Therefore, the clinical diagnosis of PCP relies mainly on the microscopic examination done after chemical or immunofluorescence staining (1). However, the sensitivity and specificity of this test are not accurate. The detection of 1,3-β-D-glucan (G test) in serum can also be used to identify *P. jirovecii* but with low specificity. Polymerase chain reaction (PCR)-based diagnostic methods have a significantly higher sensitivity and specificity, whereas the related commercial kits have not yet been approved for clinical use in China (2).

Recently, metagenomic next-generation sequencing (mNGS) has rapidly emerged as a promising single, unbiased, and culture-independent diagnostic tool and has been applied in various clinical practices (3–5). Different from other molecular assays, which can target only a limited number of known pathogens using specific primers or probes, mNGS can detect all DNA or RNA present in a sample, including the entire microbiome (3). Furthermore, mNGS is less affected by prior antibiotic exposure (6). It has been used as a diagnostic tool for various pathogens such as *Mycobacterium tuberculosis*, viruses, anaerobes, and fungi.

Here, we report the case of a 6-month-old child with PCP diagnosed by using mNGS. The clinical results of tests, including those of various antibody tests, the G test, and culture test, were all reported to be negative. An investigation revealed that the child had a previous history of *E. coli* septicemia, following which whole-exome sequencing was performed. This confirmed the presence of an X-linked immunodeficiency in the child.

## Case report

A 6-month-old male infant was hospitalized in a local hospital with recurrent fever and cough for 1 month. The presence of *E. coli* was detected by using a blood culture test. After 14 days of ceftazidime treatment, his temperature returned to normal, and the blood culture test results turned negative. However, he soon developed high fever and cough again with shortness of breath and was transferred to the West China Second University Hospital of Sichuan University. It was found that the child had developed *Klebsiella pneumoniae* septicemia during the neonatal period and pneumonia at 2 months of age. The newborn screening test results of the child were negative, and there was no evidence of any abnormalities or anomalies in the family history of close members of the child, in birth history, or in environmental history. Upon admission, a physical examination revealed a high body temperature of 39.5°C, a pulse rate of 180 beats per minute, a respiration rate of 70 per minute, and a blood oxygen saturation rate of 70%. Moist rales and wheezing were found bilaterally.

**Figure 1:**
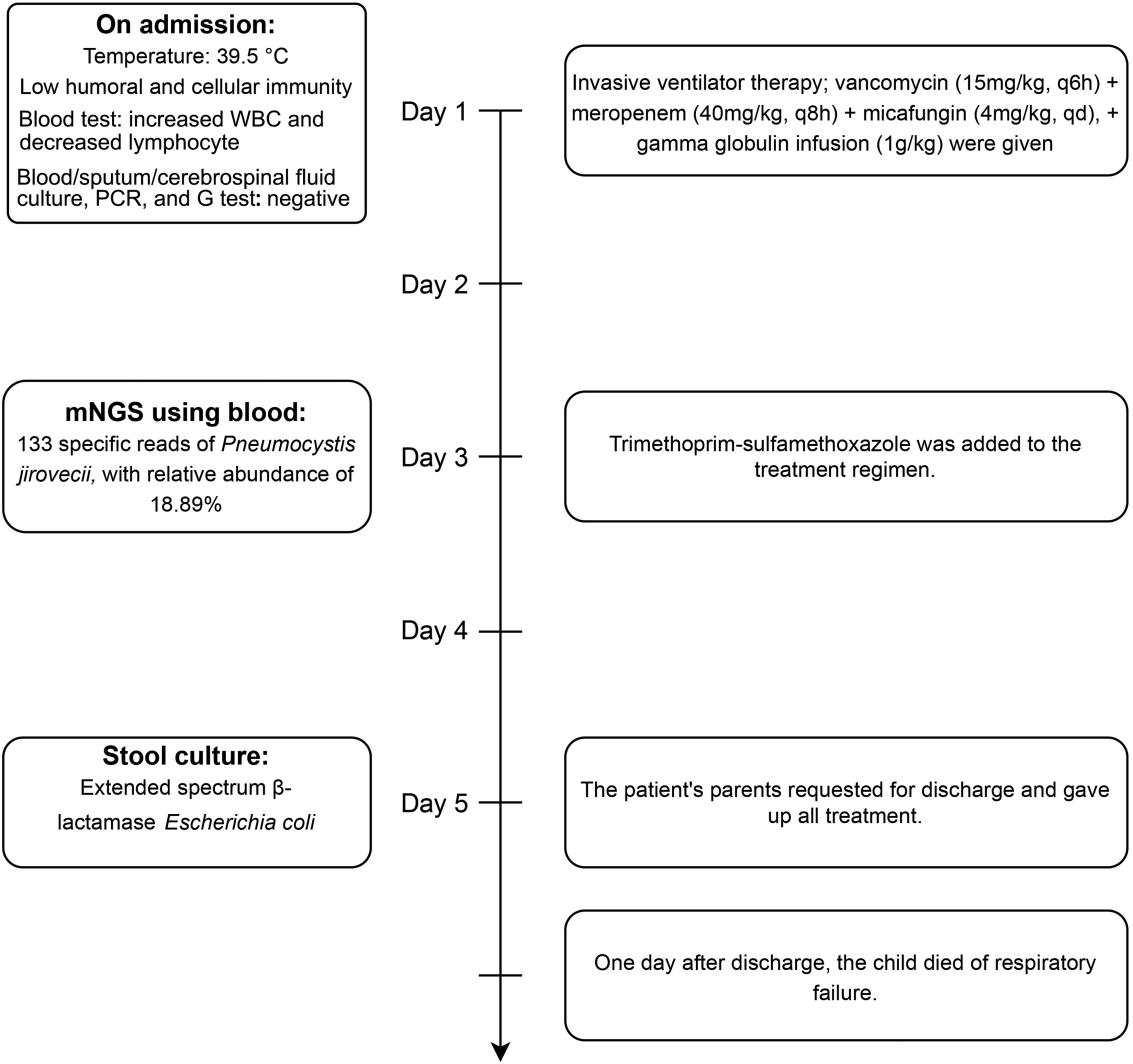
The timeline information of this patient.

**Figure 2:**
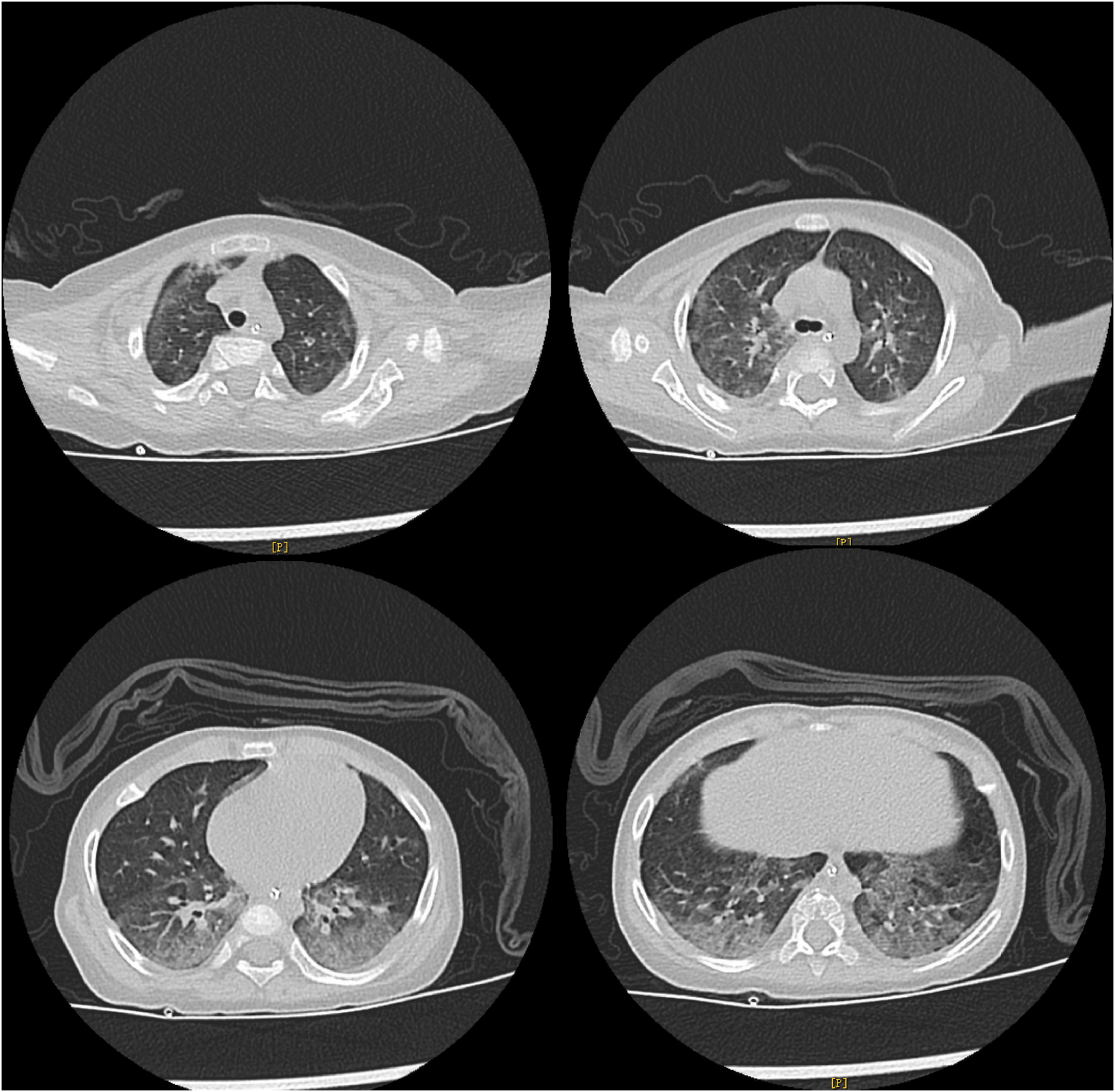
Lung CT results of the child. Lung CT shows inflammation with decreased light transmittance, which is evident in the lower lobe of both lungs, as well as the exudation shadow and strip shadow. No thymus shadow is found.

The detailed timeline of this patient is shown in Figure 1. Upon admission, laboratory auxiliary blood routine examination revealed an increased white blood cell (WBC) count (16.9 × 10^9^/L) and decreased lymphocyte absolute value (0.69 × 10^9^/L), with an absolute value of monocytes of 0.30 × 10^9^/L, absolute value of neutrophils of 15.24 × 10^9^/L, C-reactive protein of 19 mg/dl, and procalcitonin (PCT) of 8.0 ng/mL. Cellular immune response revealed a lymphocyte absolute value of 0.34 × 10^9^/L, absolute value of T lymphocytes of 0.02 × 10^9^/L, absolute value of B lymphocytes of 0.28 × 10^9^/L, and absolute value of NK lymphocytes of 0.02 × 10^9^/L. The results of culture tests performed using blood, sputum, and cerebrospinal fluid were negative. The result of the PCR test for influenza A/B virus, parainfluenza virus, *Mycoplasma pneumoniae*, *Chlamydia*, respiratory syncytial virus, metapneumovirus, rhinovirus, Bocavirus, adenovirus, and coronavirus was negative. The G test result for fungi was also negative. Lung computed tomography (CT) showed inflammation with decreased light transmittance, which was evident in the lower lobe of both lungs, as well as an exudation shadow and strip shadow (Figure 2). No thymus shadow was found (Figure 2). The results of sputum culture and two blood culture tests were also negative.

After admission, the patient received invasive ventilator therapy, anti-infective treatment with vancomycin (15 mg/kg, q6h) plus meropenem (40 mg/kg, q8h) and micafungin (4 mg/kg, qd), and gamma globulin infusion (1 g/kg). Considering the previous medical history (*E. coli* septicemia, *K. pneumoniae* septicemia, and pneumonia), lymphopenia, and the absence of thymus, congenital immunodeficiency, and opportunistic bacterial infection could not be ruled out. mNGS of blood was applied for rapid detection of pathogens. Meanwhile, whole-exome sequencing was performed in samples collected from the family members. The mNGS test results revealed 133 specific nucleic acid sequences of *P. jirovecii*, suggesting an infection with this pathogen (PCP). Trimethoprim–sulfamethoxazole was added to the treatment regimen. After 5 days of treatment, the patient's condition improved, but he still needed ventilator support. The patient's parents requested a discharge due to financial reasons and declined the provision of any further treatment for the child. A day after discharge, the child died of respiratory failure. The result of whole-exome sequencing suggested an X-linked immunodeficiency. A hemizygous mutation c.865c > t (p.r289*) was detected in the *IL2RG* gene, which was inherited from the mother (heterozygous state). The family also declined an autopsy for the child, and therefore, an anatomical diagnosis could not be obtained.

## Discussion

*Pneumocystis* pneumonia is a major opportunistic infection in immunocompromised patients (7). The increasing rates of incidence of this disease seriously threaten global public health and economy. In an 11-study meta-analysis that evaluated the performance of the G test for *P. jirovecii*, an overall sensitivity rate of 95% (95%CI: 91%–97%) was found (8). However, in our hospital, the G test does not exhibit the same high level of sensitivity for the diagnosis of PCP. mNGS is a state-of-the-art technology that has been increasingly applied in clinical practice for various infectious diseases such as pneumonia and sepsis (3). It can rapidly detect almost all pathogens in the specimens and is often more sensitive and specific for diverse infections than conventional methods, especially for pathogens that are difficult to diagnose and for patients with prior antibiotic exposure (9).

In this case, the child developed acute pneumonia and sepsis. He had previously suffered from *E. coli* septicemia, *K. pneumoniae* septicemia, and pneumonia. Multiple antibiotics had been given, which made the diagnosis by some conventional methods difficult. This suggests that mNGS has a significant advantage over conventional methods in detecting *P. jirovecii*, especially when a patient has received antibiotics treatment previously.

In addition, the child had experienced various infections in the past 6 months, which is uncommon. This made us to consider the immunodeficiency of the child. Whole-exome sequencing was performed and a hemizygous mutation c.865c > t (p.r289*) was found in the *IL2RG* gene. The *IL2RG* gene encodes the common chain (γc) of several interleukin receptors, which are mainly involved in the body's immune process (10). This mutation can cause an X-linked severe combined immunodeficiency, a profound deficiency of T, B, and natural killer cell immunity. Patients with X-linked severe combined immunodeficiency can commonly develop candidiasis, pneumonia, bronchitis, and other repeated infections within the first few months of birth, along with growth retardation due to gastrointestinal malabsorption (11). The child in this case was finally diagnosed with PCP and X-linked immunodeficiency inherited from the mother.

In conclusion, this case report highlights the value of mNGS in diagnosing PCP when conventional diagnostic methods fail to identify the agent. Early onset of recurrent infectious diseases suggests the possibility of immunodeficiency disease. Early genetic analysis and diagnosis is necessary for patients with such disease conditions.

## Data Availability

The datasets presented in this study can be found in online repositories. The names of the repository/repositories and accession number(s) can be found in http://ngdc.cncb.ac.cn, PRJCA014514.
